# Acute Onset Postpartum Pleural Effusion: A Near-Miss Maternal Case Due to Scrub Typhus Infection

**DOI:** 10.7759/cureus.35142

**Published:** 2023-02-18

**Authors:** Neema Acharya, Sourya Acharya, Samarth Shukla, Jyotsana Potdar, Tejal Waghe, Ruchita Kabra

**Affiliations:** 1 Department of Obstetrics and Gynaecology, Datta Meghe Institute of Health and Education Research (DMIHER), Wardha, IND; 2 Department of Medicine, Datta Meghe Institute of Health and Education Research (DMIHER), Wardha, IND; 3 Department of Pathology, Datta Meghe Institute of Health and Education Research (DMIHER), Wardha, IND

**Keywords:** pleural effusion, scrub typhus, endemic, peripartum, puerperal

## Abstract

A 32-year-old puerperal patient developed acute onset breathlessness and fever on the third postoperative day. On evaluation, the patient was diagnosed to have scrub typhus pneumonia without any characteristic eschar. The condition was associated with pleural effusion, and it was drained. Azithromycin was used as the drug of choice due to the peripartum status of this patient. The patient improved due to early detection and multidisciplinary timely care. The safe outcome of this near-miss case suggests that fever profile workup, especially in scrub typhus endemic areas, should include scrub typhus testing even if classical signs are absent in the peripartum period.

## Introduction

Scrub typhus in pregnancy is a rare infection that an obstetrician may come across. Though rare, it can lead to multiorgan failure and catastrophic feto-maternal outcomes [1]. Scrub typhus is commonly found in Asia and is caused by the intracellular bacteria *Orientia tsutsugamushi* via a flea vector [2]. Most of the cases reported in India belong to the hilly Himalayan area [3,4]. Very few cases have been reported from non-hilly areas of central India. Presenting herewith a near-miss case of bilateral interstitial pneumonitis in a post-operative case of cesarean section.

## Case presentation

A 32-year-old second gravida with a history of previous cesarean section presented in labor. She was a dairy farm worker by occupation. Emergency ceserean section was done as intrapartum; the liquor was meconium stained, and fetal cardiotocography showed baseline bradycardia, and the immediate postoperative course was uneventful for 48 hours. She was breastfeeding the child. On day three postoperatively, the patient developed high-grade intermittent spikes of fever and breathlessness with a non-productive cough.

On examination, she was febrile (102 degrees Fahrenheit), pulse was 130/ minute, regular. She was tachypneic (respiratory rate 28/min), with a blood pressure of 100/70 mm of Hg and SpO2 of 90% while breathing ambient air. Respiratory system examination revealed coarse crepitations in bilateral lung fields and a stony dull note with absent air entry in the left infraaxillary and infra-scapular area. The abdomen was soft, bowel sounds were present, and the suture line of the cesarean section was healthy. Lochia was healthy, and her breasts were not engorged.

**Figure 1:**
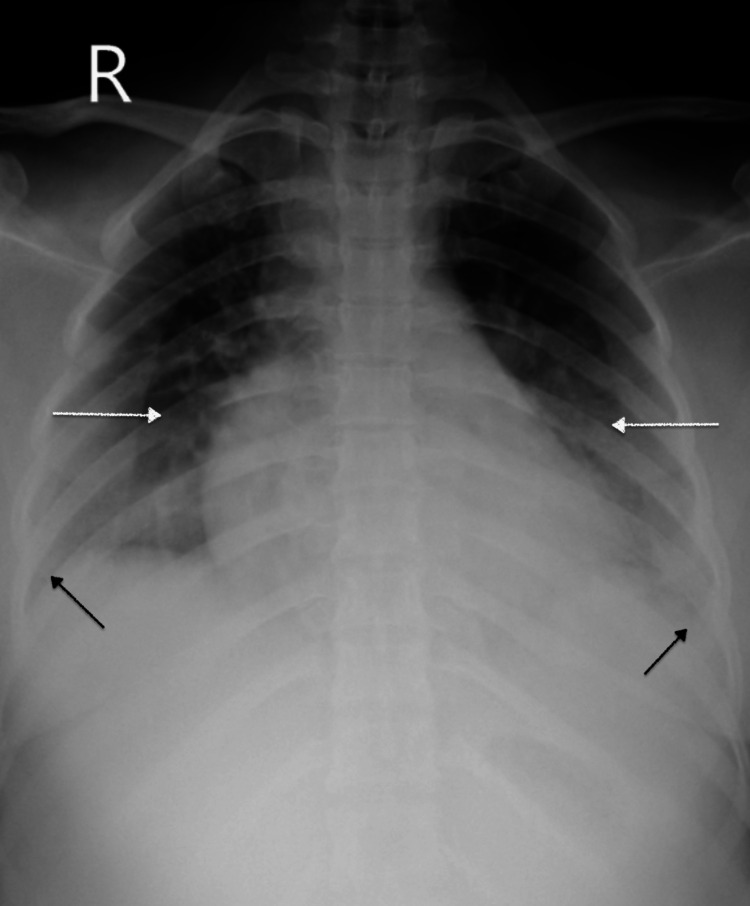
Chest X-ray showing bilateral consolidation (white arrows) with blunting of costophrenic angles (black arrows) suggestive of effusion.

The patient was shifted to the intensive care unit for observation and further management. Chest X-ray revealed bilateral consolidation with bilateral parapneumonic effusions left more than right (Figure 1).

Her blood investigations revealed hemoglobin of 11.2gm%, white blood cells of 13,500/cumm with predominant neutrophils, platelet count of 1.8 lakhs, hematocrit 40%, C-reactive protein of 2.5mg/dl, ESR (erythrocyte sedimentation rate) 22mm/hour, LDH (lactate dehydrogenase)- 250IU/L, serum protein 45gm/dl. Peripheral smear for malarial parasite was negative. Also, Immunoglobulin M (IgM), Immunoglobulin G (IgG), and nonstructural protein 1 (NS1) antibodies for dengue, IgM, and IgG antibodies for Leptospira were negative, but IgM antibody for scrub typhus was positive. Liver and kidney function tests were within normal limits. Antibodies for scrub typhus were done as this area is endemic for scrub typhus. As the antibody for scrub typhus was positive, the patient was examined for eschar on the body, but it was not visible.

**Figure 2:**
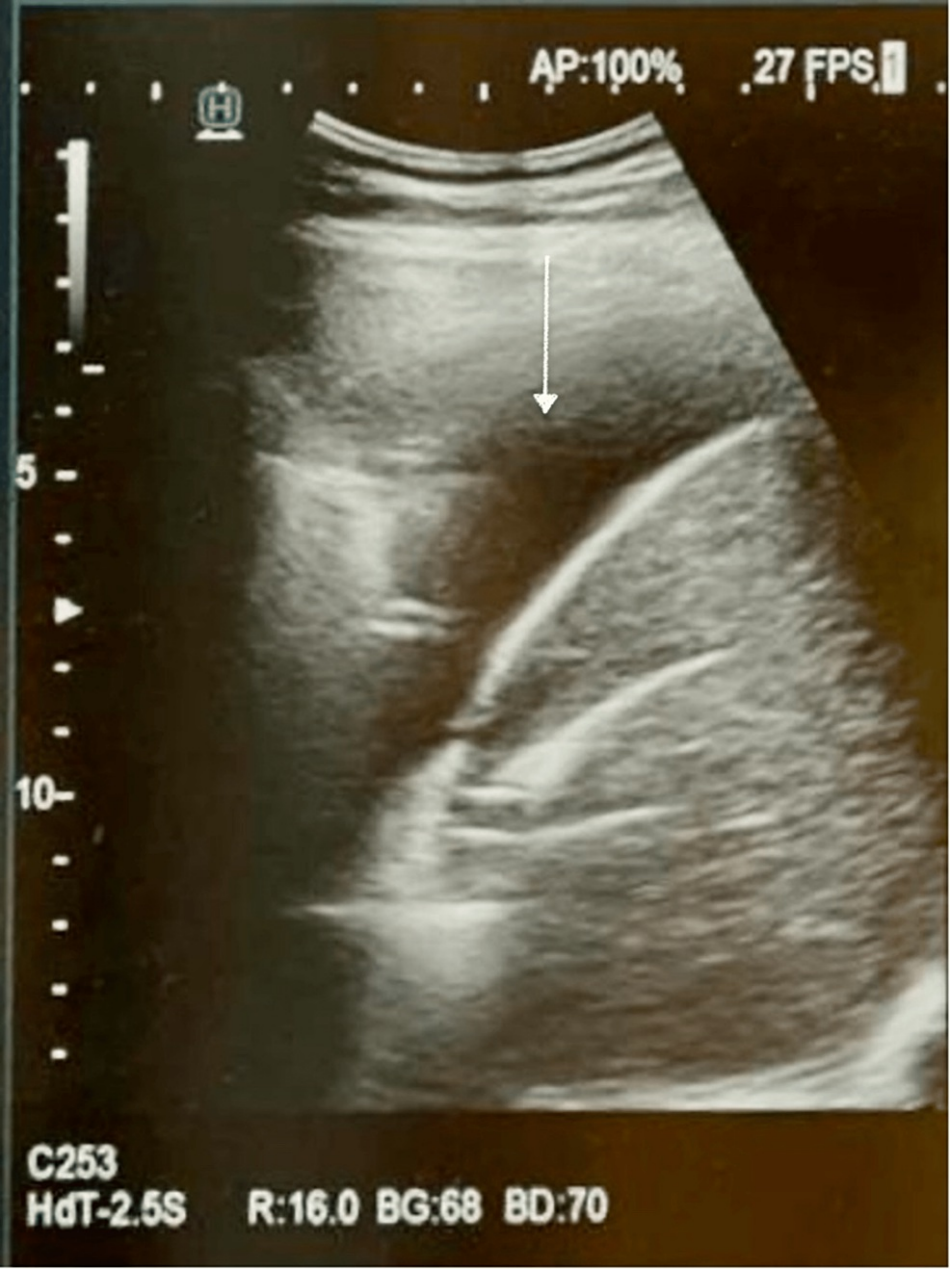
Ultrasonography of the thorax showing pleural effusion with subsegmental lung collapse on the left side (white arrow).

She was started on supplemental oxygen therapy. Due to the peripartum status tablet of azithromycin, 500 mg once a day for five days was added as the preferred drug to postoperative antibiotic prophylaxis. Ultrasound of the thorax suggests pleural effusion with subsegmental lung collapse on the left side, as shown in Figure 2.

Ultrasound-guided drainage of pleural fluid was done from the left pleural cavity, and 500 ml of pleural fluid was drained; pleural fluid analysis revealed total white blood cells- 55, proteins- 21gm/dl, glucose- 32mg/dl, LDH (lactate dehydrogenase)- 120IU/L which suggestive of exudative effusion by Light's criteria. Pleural fluid ADA (adenosine deaminase) was negative. The patient was afebrile after three days, breathlessness subsided, and the drain was removed after seven days. SpO2 was 98% in room air. The rest of the post-operative period and wound healing were uneventful.

During critical care days, the baby was fed through a milk bank and was isolated from the mother. The baby was investigated for a scrub typhus antibody test and was negative. The patient was discharged in good general condition.

## Discussion

Acute febrile fever following surgery is not unusual. Scrub typhus is rarely considered the cause of fever, interstitial pneumonia, and pleural effusion. Although scrub typhus is typically associated with mountainous regions, in this instance, it may also be found in Indian plains. Scrub typhus manifests abnormally during the peripartum period because of the body's altered immune response mechanism. In patients with scrub typhus, presenting late or with multiple organs, involvement increases mortality [5,6].

A case series reports analysis of six pregnant and two puerperal women having scrub typhus infection reported that none of them showed normal lymphadenopathy and eschar. However, research from south India found that eschar was identified in almost half of the reported scrub typhus cases. In this case report, the eschar was not visible [7,8]. The ICU requirement, higher APACHE scores, low albumin levels, and absence of eschar are all indicators of a poor prognosis in such cases [9].

Interstitial pneumonia and vasculitis are the primary pathologic mechanism in respiratory system involvement in scrub typhus infection, which is extensively reported. Patients may exhibit lung involvement in up to 58.4% of cases, evident with symptoms causing cough and dyspnoea. Acute respiratory distress syndrome affects roughly 11.1% of these patients and reportedly shows a high fatality rate of 25%, which is a significant and dangerous sign of scrub typhus. One recent study from India shows ARDS incidence at 19.2% with a higher fatality rate (33%). In 12% to 55% of individuals, pleural effusion is seen [10].

Some authors consider the presence of interstitial pneumonia as a marker for predicting the clinical course and prognosis because it is associated with a more serious clinical presentation, prolonged indoor care, and a higher fatality when compared to patients who did not develop this complication. Interstitial pneumonitis with pleural effusions was our patient's initial diagnosis [11,12].

The intracellular gram-negative bacteria that caused severe vasculitis affects the lungs, heart, liver, kidneys, brain, and other essential organs. The same pathophysiology of the condition is explained in this case report also [13,14]. A high index of suspicion can detect this infection, and timely management can be started. Scrub typhus is frequently characterized by fever, myalgia, a maculopapular rash, and eschar from a vector bite [2]. As a safe peripartum medicine, azithromycin was given to this woman as a drug of choice [15]. Early detection and timely multidisciplinary management of this near-miss case prevented maternal mortality.

## Conclusions

Scrub typhus infection though rare is commonly found in endemic areas. Although there are more reports of scrub typhus in pregnant women, it is still underreported because of low awareness and the non-availability of diagnostic methods. Scrub typhus must be ruled out in pregnant females when they present with fever and respiratory symptoms. Timely diagnosis and therapy can prevent severe complications and high mortality related to scrub typhus infection in the peripartum period. 
